# Ultra-Fast Glyco-Coating of Non-Biological Surfaces

**DOI:** 10.3390/ijms17010118

**Published:** 2016-01-16

**Authors:** Eleanor Williams, Katie Barr, Elena Korchagina, Alexander Tuzikov, Stephen Henry, Nicolai Bovin

**Affiliations:** 1 Centre for Kode Technology Innovation, Faculty of Design and Creative Technologies, Auckland University of Technology, Private Bag 92006, Auckland 1142, New Zealand; ewilliam@aut.ac.nz (E.W.); katielbarr@yahoo.co.uk (K.B.); 2 Laboratory of Carbohydrate Chemistry, Shemyakin Institute of Bioorganic Chemistry, Moscow 117997, Russia; ellyu@yandex.ru (E.K.); tuzikov@carb.ibch.ru (A.T.)

**Keywords:** function-spacer-lipid, blood group A, nanofibres, surface-coating, glyco-coating, glyco-landscape, shear stress

## Abstract

The ability to glycosylate surfaces has medical and diagnostic applications, but there is no technology currently recognized as being able to coat any surface without the need for prior chemical modification of the surface. Recently, a family of constructs called function-spacer-lipids (FSL) has been used to glycosylate cells. Because it is known that lipid-based material can adsorb onto surfaces, we explored the potential and performance of cell-labelling FSL constructs to “glycosylate” non-biological surfaces. Using blood group A antigen as an indicator, the performance of a several variations of FSL constructs to modify a large variety of non-biological surfaces was evaluated. It was found the FSL constructs when optimised could in a few seconds glycosylate almost any non-biological surface including metals, glass, plastics, rubbers and other polymers. Although the FSL glycan coating was non-covalent, and therefore temporary, it was sufficiently robust with appropriate selection of spacer and surface that it could capture anti-glycan antibodies, immobilize cells (via antibody), and withstand incubation in serum and extensive buffer washing, making it suitable for diagnostic and research applications.

## 1. Introduction

Glycosylation of biological surfaces is well established and known to have important roles and the mimicking of these glycosylation patterns on non-biological surfaces has uses and potential in basic research as well as techniques ranging from medical applications [1] through to diagnostics [2]. Chemical glycosylation of surfaces usually involves covalent immobilisation of glycans onto membrane surfaces utilising a variety of coupling reactions [3]. Even enzymatic glycosylation requires chemical coating with a glyco-primer [3]. However, a few researchers have used physical adsorption of lipid-linked oligosaccharides onto membranes, as originally used by Feizi and co-workers [3]. We report here an extension of this physical adsorption method to rapidly coat almost any non-biological surface by using function-spacer-lipid (FSL) constructs previously used for the modification of cells and viruses [4,5,6,7].

FSL constructs unlike other lipidated glycans and neoglycolipids have a spacer included in their architecture. This spacer facilitates conjugation of the glycan to the lipid tail and can also be designed to bring additional features to the construct, including controlled spacing away from a membrane, ligand spacing and enhanced attachment and retention on biological and non-biological surfaces [4,5]. Unlike in the plasma membrane of a cell where the lipid tail of the FSL construct is able to insert into the lipid bilayer, on solid non-biological surfaces it instead imparts on the FSL construct an amphiphatic character, which drives the self-assembling process on surfaces and probably their surface adhesion via water-exclusion [4].

**Figure 1:**
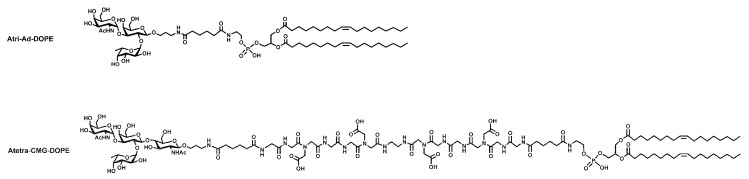
Schematic diagrams of the two primary blood group A function-spacer-lipid (FSL) constructs used in this paper. The upper schematic Atri-Ad-DOPE shows an FSL with a trisaccharide generic blood group A antigen and a short 2 nm adipate spacer while the lower schematic Atetra-CMG-DOPE shows a type 2 chain specific A tetrasaccharide FSL with a longer 7 nm carboxymethylglycine spacer. Both constructs have the same dioleoylphosphatidylethanolamine (DOPE) lipid tail.

There are a large range of glyco-FSL constructs and many have been shown to have biological applications [4,5]. For this study, two primary FSL construct variants (Figure 1) were chosen, one based on the short 2 nm adipate spacer (Atri-Ad-DOPE) and the other on the longer 7 nm carboxymethylglycine spacer (Atetra-CMG-DOPE). Techniques for the visualization of these specific constructs are well-established [4,5,8]. Using these two constructs, each with potentially very different attributes, we examined the performance of FSL constructs to modify a range of non-biological materials.

## 2. Results and Discussion

### 2.1. Surface Variations

#### 2.1.1. Surface Variations—Coupons

**Figure 2:**
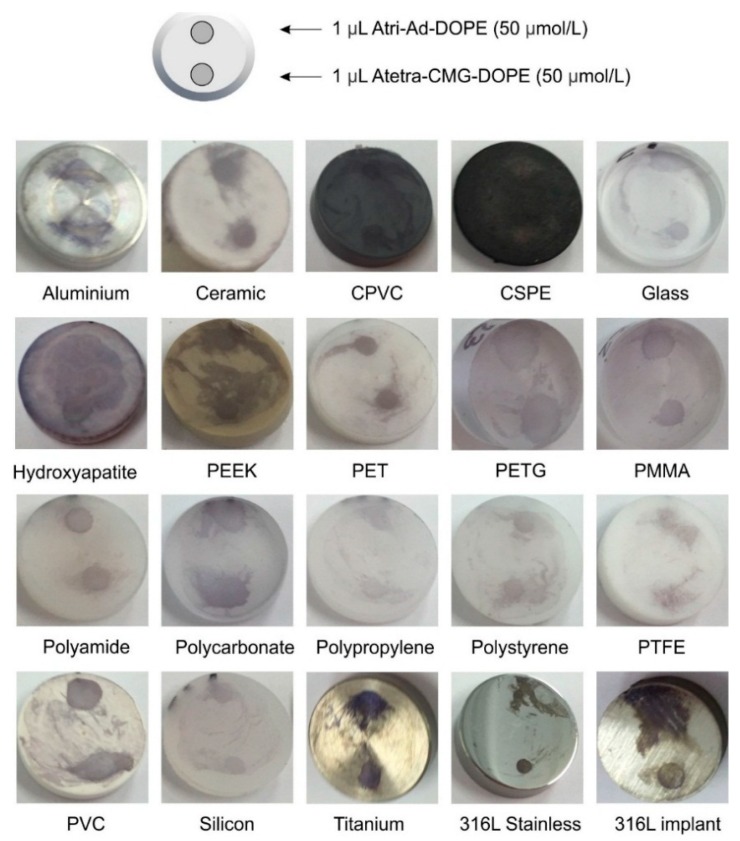
Photographic images of 20 representative coupons (see also Table 1) each spotted with 1 µL of Atri-Ad-DOPE (12 o’clock spot) and Atetra-CMG-DOPE (6 o’clock spot). Schematic diagram shows experimental layout. FSL spotted coupons were allowed to air dry then visualised by EIA using monoclonal anti-A and precipitating chromogenic substrate (purple).

A variety of standardized materials in the form of coupons were labelled with both Atri-Ad-DOPE and Atetra-CMG-DOPE (Table 1 and Figure 2). With the exception of those surfaces that degraded (corroded) under experimental conditions (e.g., iron, copper), all surfaces were labelled with both FSL constructs. The relative surface areas of the coupons and their ability to retain the enzyme immunoassay (EIA) precipitate potentially contributed to the variations in intensity seen between different materials. Additionally, it is possible that on some surfaces the development of the chromogenic precipitate may have also been inhibited to some degree by (electro) chemical activity of the surface.

When comparing the results between the two different FSL constructs on the same surface there were often differences in their ability to remain as the discrete spot originally applied. In general, on metallic surfaces, the Atri-Ad-DOPE construct often smeared across the coupon. This smearing probably occurred during the first EIA washing step. The most extreme smearing occurred with Atri-Ad-DOPE on hydroxyapatite where this FSL construct spread almost over the entire coupon, while the Atetra-CMG-DOPE on the same coupon remained as a discrete spot. In general plastics, rubbers and other polymers are labelled similarly with both constructs, although there were differences between materials. For example, both FSL constructs smeared on PEEK, CPVC, polycarbonate and PTFE, while Atetra-CMG-DOPE appeared to smear more on polystyrene and PVC than did Atri-Ad-DOPE. These surface differences would be expected due to the different surface characteristics including charge and hydrophobicity and relative abilities to interact with different zones of the FSL constructs. Despite the differences in surface composition both FSL construct variations were able to adhere to all surfaces tested (Table 1).

**Table 1:** Summary of surfaces successfully coated with Atri-Ad-DOPE and Atetra-CMG-DOPE constructs (with examples shown in Figure 2 and Figure 4).

Surface	Alphabetical Listing of Materials Modified by Blood Group A Fsl Constructs
Metals	Aluminum, Copper, Gold, Nickel, silver, Stainless Steel (304), Stainless Steel (316L), Stainless Steel (347), Titanium
**Plastics/Polymers/Rubbers/Fibres (Alphabetical Order)**	Acrylonitrile butadiene styrene (ABS), Cellulose acetate (transparency film), Cellulose acetate (nanofibres), Chlorinated polyvinyl chloride (CPVC), Chlorosulfonated polyethylene (CSPE, hypalon), Cotton, Ethylene propylene diene monomer (EPDM) rubber, Mixed cellulose esters, Natural rubber, Nitrile butadiene (NBR) rubber, Nitrocellulose, Poly(methyl methacrylate) (PMMA: Plexiglass), Polyamide (Nylon), Polyamide PA66 nanofibres, Polycarbonate, Polyetheretherketone (PEEK: Arlon 1330), Polyethylene terephthalate (PET: Polyester, Dacron), Polyethylene terephthalate glycol (PETG), Polyethylene UMHW, Polypropylene, Polystyrene, Polytetrafluoroethylene (PTFE), Polyurethane (high temperature polymer), Polyvinyl butyral nanofibres (PVB), Polyvinyl chloride (PVC), Polyvinylidene fluoride (PVDF), Regenerated cellulose, Silicone rubber, Silk, Silica gel S60 (TLC plate), Silica gel C18 (TLC plate), Viton rubber, wood (various)
Other	Borosilicate glass, Concrete, Ceramic tile-glazed, Hydroxyapatite, Ceramic porcelain, Paper-24 varieties of coated and uncoated papers

#### 2.1.2. Surface Variations—Printing and Direct Application

**Figure 3:**
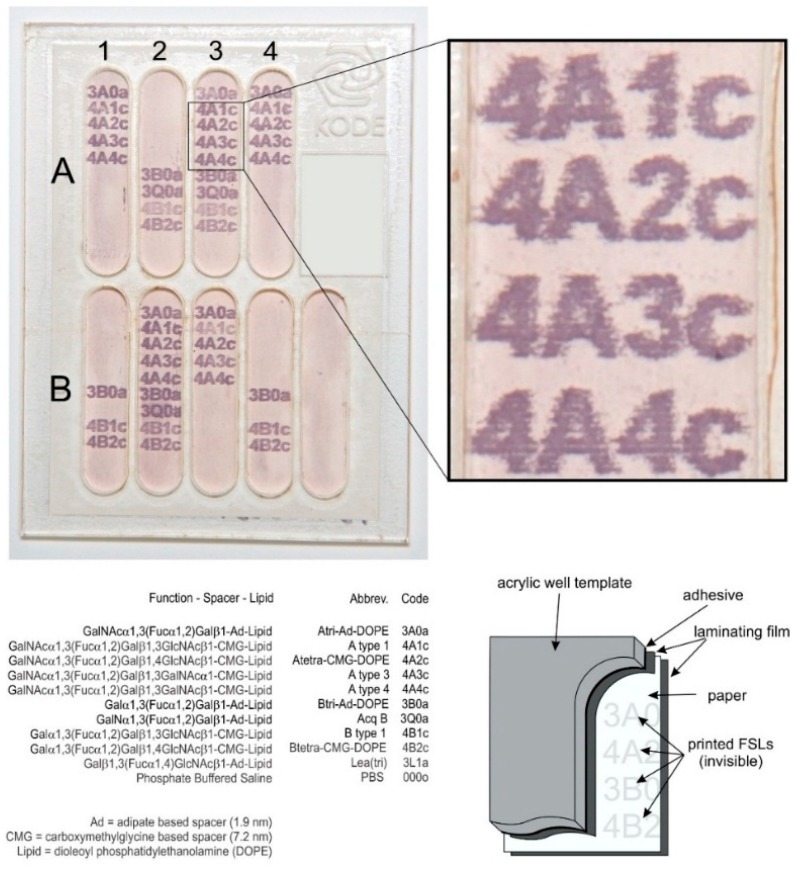
An example of microplate prepared with blood group **A** and **B** FSL constructs printed on paper (MG paper—cellulose esters) and prepared as a microplate to determine specificity of monoclonal reagents. Reprint from [8]. Copyright 2014 with permission from John Wiley and Sons. Alphanumeric characters appear when developed in the EIA reaction wherever the monoclonal reagent has bound to the FSL. Area outside of the printed area is the “internal” negative control and/or blank. In this example antibodies in co-ordinates **A1**, **A4**, **B3** have anti-A specificity, those in **A2**, **B1** and **B4** have anti-B specificity while those in **A3** and **B2** anti-A + B specificity. This EIA technique either with printed alphanumeric characters or spots of FSL constructs on a variety of different surfaces and optionally prepared as microplates was the basis for most experiments.

In addition to disc coupons, a variety of other surfaces were also available (Table 1). Those available in planar form were able to have their blood group A FSL coating applied with an inkjet printer (Figure 3 [8]). Inkjet printing of the FSL constructs was particularly valuable as it also provided discrete and identifiable borders of where the FSL had been applied, thus allowing for observations of the degree of smearing, or “bleed”. Furthermore, because the FSL could be printed as identifying information it was able to facilitate sample identification (Figure 3). Planar surfaces could also be constructed into microplates (Figure 3 [8]).

**Figure 4:**
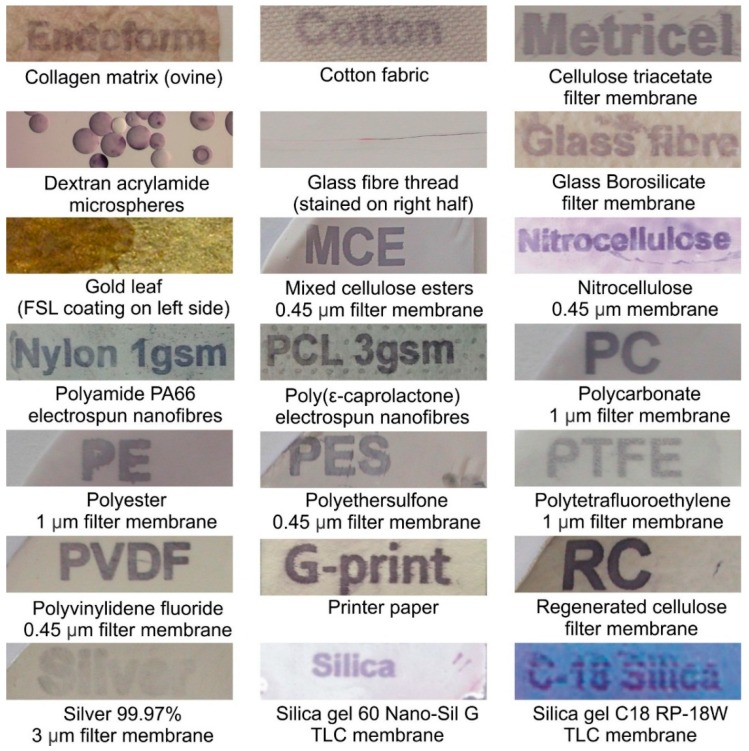
Examples of 21 different surfaces reacted with Atri-Ad-DOPE. FSL constructs on most surfaces were inkjet printed as words identifying each surface material. The microspheres and glass fibre were coated with FSL construct by immersion, while a paintbrush was used to apply the FSL construct to gold foil. The blood group A FSL constructs bound to the various surfaces were visualised by EIA using monoclonal anti-A and a precipitating chromogenic substrate.

A variety of planar surfaces were printed with Atri-Ad-DOPE and stained by EIA (Figure 4). Non-inkjet printable surfaces such as microspheres, glass fibres threads and gold foil were labelled by application of the FSL to their surface by either immersion or application with a paintbrush. No surface was found that was unable to be FSL modified, and although some surfaces appeared to stain stronger than others, this was probably due to differences in surface area, organization of adhered FSLs, and ability of the EIA precipitate to remain at the surface.

#### 2.1.3. Cell Adhesion to Printed FSL Constructs

**Figure 5:**
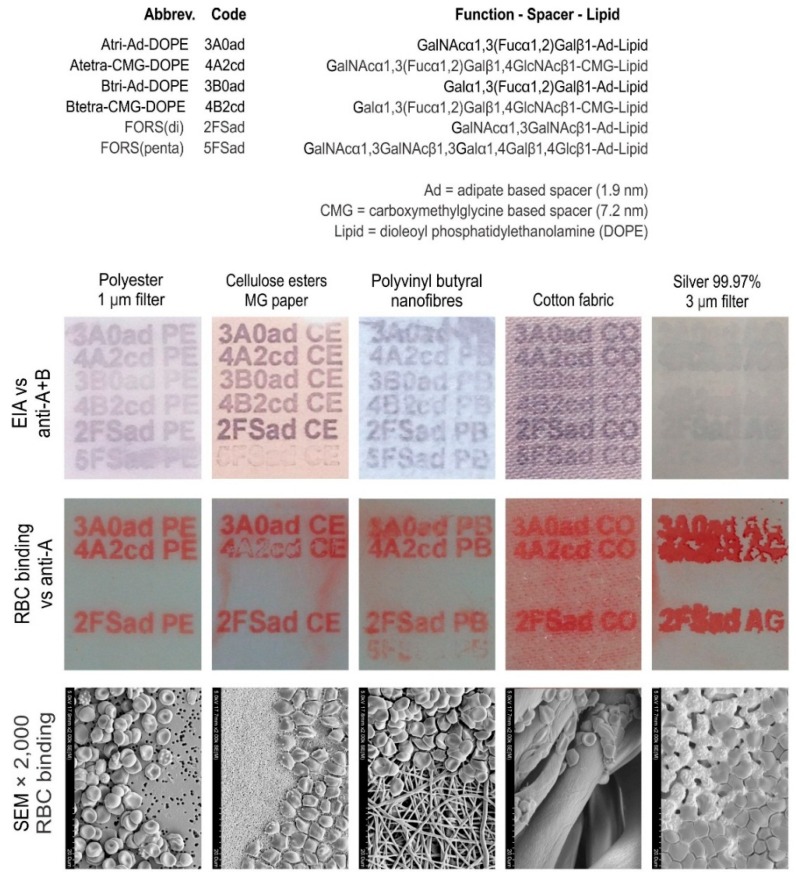
Capture of red blood cells onto printed glyco-FSLs. Six different ABO related FSL constructs based on adipate and CMG spacers were printed onto five different surfaces. The upper row of images show the presence of these FSL constructs on surfaces using EIA and monoclonal antibodies directed against both the A and B antigens. The middle row of images show blood type specific binding of these same surfaces when reacted with monoclonal IgM anti-A and used to capture blood group A red cells. Of particular note is the poor EIA and strong red cell binding reaction of the silver membrane, highlighting the inability of some surfaces to show strong reactivity by the EIA method. The lower row of images are SEM 2000× magnifications of the edge of the printed areas, showing delineation between the printed blood group A FSL + IgM anti-A capture of blood group A red cells area and unprinted areas. It can be seen by its ability to immobilise RBCs that the monoclonal anti-A used had higher cross reactive affinity for the Forssman disaccharide (2FSad) than for the pentasaccharide (5FSad). This is expected as the Forssman antigen is a cross-reactive target of the anti-A reagent used, and the pentasccahride with a more complete Forssman structure will have less off-target binding [9].

The binding of red cells can also be used to detect the presence of constructs. In this scenario rather than using an alkaline phosphatase labelled anti-immunoglobulin to detect the primary IgM antibodies (Ab) bound to the blood group A constructs as would be done in the EIA, the IgM anti-A bound to blood group A FSL is used to capture blood group A red cells. As seen in Figure 5 a variety of blood group A, B and related FSL antigens were printed onto different surfaces with an inkjet printer. Immunostaining of these FSLs with an antibody mix (anti-A + anti-B) that reacts with all constructs reveals their presence (although the silver filter membrane appears to be poorly reactive). When these surfaces, after reacting with IgM anti-A, are overlaid with blood group A red cells (instead of a secondary anti-Ig enzyme conjugate as in the EIA), the red cells bind very strongly and specifically to blood group A FSL/Ab complex areas on the surface. Subsequent experiments (not shown) demonstrate that the enzyme precipitate produced in the EIA reaction is retained very poorly on some surfaces like silver, despite significant FSL binding (as visualised by cell capture). Not only does this experiment show an alternative method to visualise constructs on some surfaces (particularly metals) but also that the binding of the FSL to a surface is sufficient to immobilise cells under low shear forces. If these surfaces with captured cells are subjected to higher shear forces (e.g., washing or agitation) then the red cells will be dislodged from the surface.

#### 2.1.4. Microsphere Binding

**Figure 6:**
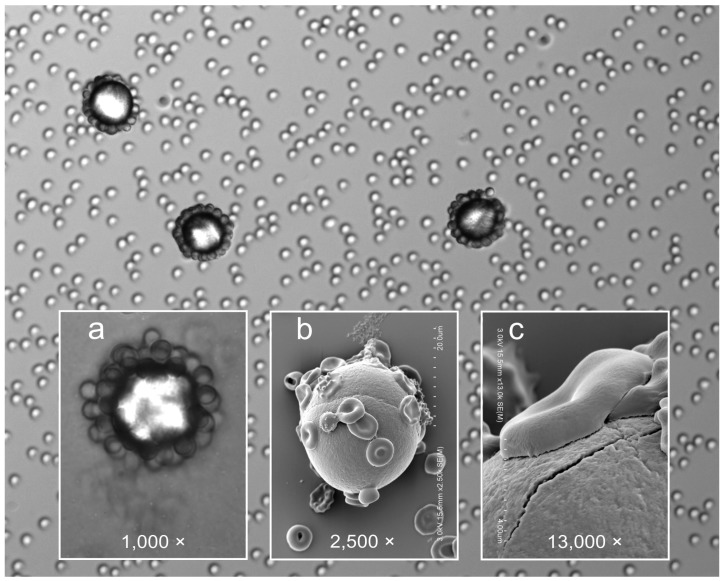
Attachment of blood group A red cells onto 20 µm polycarbonate microspheres coated with Atri-Ad-DOPE and IgM anti-A. Inset (**a**) shows light microscopy image at 1000× magnification while insets (**b**) & (**c**) show higher magnifications under SEM (post glutaraldehyde fixation). Although the main image and inset (**a**) appears to show cells only on the perimeter of the microsphere, this is an artefact due to the microscopic plane of focus. Varying the plane of focus reveals red cells evenly distributed over the microspheres. No binding occurred on controls.

If the FSL constructs are applied to microspheres (by simple contact), and these FSL modified microspheres are then coated with IgM antibody (and washed), these microspheres are able to specifically capture antigen positive cells (Figure 6). The SEM image (Figure 6, inset c) appears to show the bound red cells distorting and having firm contact with the modified microsphere surface. However the interaction is not robust, and captured red cells can be released from the microspheres by subjecting them to shear forces such as agitation (e.g., vortex) or aggressive washing procedures. A range of different microspheres including paramagnetic beads [10] have been modified with FSL constructs.

#### 2.1.5. Nanofibre Incorporation

**Figure 7:**
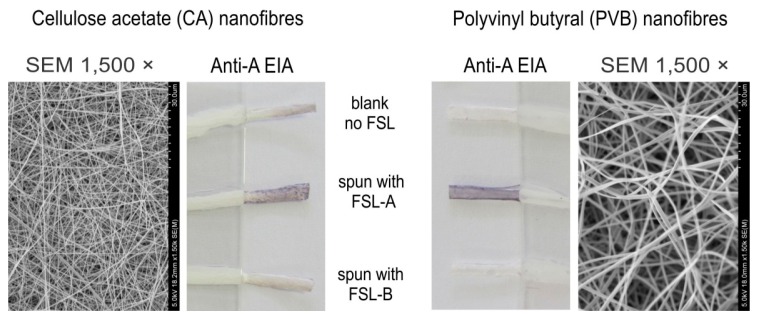
Incorporation of Atri-Ad-DOPE into nanofibres during the electrospinning process. Cellulose acetate (CA) and polyvinyl butyral (PVB) nanofibres were electrospun with Atri-Ad-DOPE and Btri-Ad-DOPE constructs included in the pre-spinning liquid polymers. Spun nanofibres were twisted into cords and then immunostained. Staining with monoclonal anti-A by the EIA method was able to demonstrate incorporation of the blood group A FSL construct into both nanofibres during the electrospinning process.

The ability of the FSL constructs to self-assemble on nanofibres during the electrospinning process was established by adding the Atri-Ad-DOPE construct to two different nanofiber polymers (CA and PVB) before electrospinning of the fibres [11]. The resulting fibres when stained with the EIA technique shows incorporation of the FSLs into the fibres, and presence of the immunoreactive antigen at the nanofibre surface (Figure 7). No disruption in the formation or structure of the nanofibres was observed when the FSL constructs were included in the polymers.

### 2.2. Binding Characteristics and Performance

#### 2.2.1. Molarity

**Figure 8:**
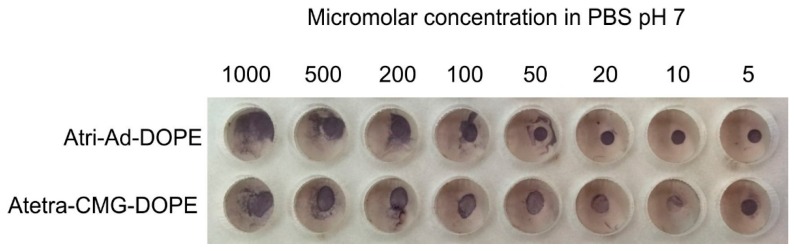
Effect of molar concentration on the surface binding characteristics of Atri-Ad-DOPE and Atetra-CMG-DOPE when applied as a 1 µL spots on MG paper.

In order to determine the molar range in which Atri-Ad-DOPE and Atetra-CMG-DOPE would produce detectable EIA reactions a series of dilutions (in PBS pH 7) of the FSLs was prepared over the range of 5–1000 µmol/L and contacted with paper for 5 min, washed and allowed to dry. As can be seen in Figure 8 the 1 µL spots of both constructs were easily detectable at 5 µmol/L. Of note was that with concentrations greater than 50 µmol/L smearing of the original spot occurred. This is probably due to layering of the FSL constructs upon themselves, and with these multilayers being dissociated during the washing process. As the time of contact for an FSL to adhere to a surface can be extremely rapid (see below) the loosely bound FSL’s released during the washing process probably immediately bound to unmodified surface and remained adhered. It is also of note that the Atri-Ad-DOPE construct appeared to bleed more than the Atetra-CMG-DOPE construct, an observation concordant with those seen before (Figure 2). This suggests that potentially the CMG based FSL constructs are able to interact more strongly with each other than the adipate based constructs.

#### 2.2.2. pH

**Figure 9:**
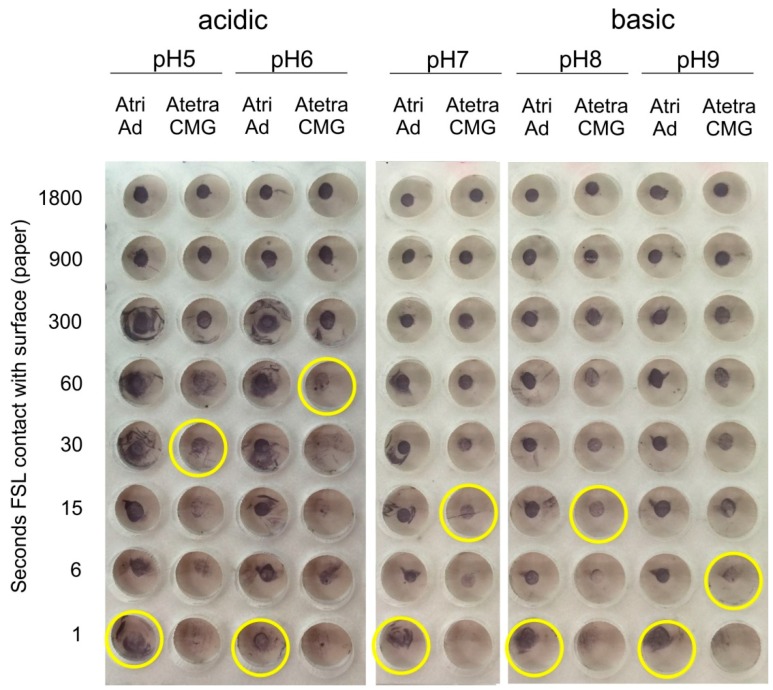
Effect of application buffer pH on surface binding characteristics of Atri-Ad-DOPE and Atetra-CMG-DOPE when applied as a 1 µL spots (50 µmol/L) on paper. Columns show the time of contact in seconds of the FSL with the surface before being washed with PBS. The last clearly positive reaction (defined as even reactivity over the spot area) in each column is indicated by a yellow circle.

The effect of pH was studied by preparing 50 µmol/L Atri-Ad-DOPE and Atetra-CMG-DOPE in a series of 0.1 M phosphate buffers over the range of pH 5–9. These pH adjusted FSL constructs were applied to MG paper and allowed to be in contact with the surface for between 1 and 1800 s minutes (30 min) before being washed in PBS pH 7. Binding of the FSL constructs to the surface was then determined by EIA (Figure 9).

It can be seen that although binding of both constructs were largely unaffected by acidic pH the Atetra-CMG-DOPE required more time (30–60 s) to get good binding to the paper surface, compared with 1 s for Atri-Ad-DOPE. The binding of Atri-Ad-DOPE constructs appeared to be indifferent to pH over the range of 5–9 while the Atetra-CMG-DOPE construct appeared to have improved binding at higher pH (e.g., 30 s at pH 5 compared with 6 s at pH 9). These results possibly relate only to MG paper and performance on other surfaces (and with different concentrations of FSL constructs and ionic strength) could potentially produce different results to those obtained on paper. See also results below relating to surface differences observed at the same pH or with different application buffer/solvent.

#### 2.2.3. Ionic Concentration

**Figure 10:**
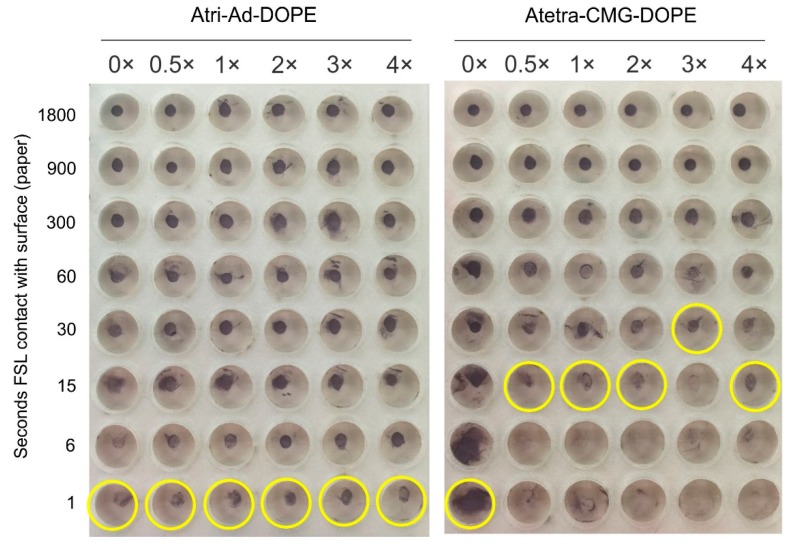
Effect of application buffer ionic concentration on the surface binding characteristics of Atri-Ad-DOPE and Atetra-CMG-DOPE when applied as a 1 µL spots (50 µmol/L) on paper. Columns show the time of contact in seconds of the FSL with the surface before being washed away with PBS. The last clearly positive reaction in each column is indicated by a yellow circle.

The effect of ionic strength of the application buffer was studied by preparing 50 µmol/L Atri-Ad-DOPE and Atetra-CMG-DOPE in a pH 7 phosphate-citrate buffer and then adjusted the ionic strength. The original isotonic buffer (1×) was adjusted by diluting 1:1 with water to produce the 0.5× buffer, and sodium chloride was added to produce 2×, 3×, 4× more salt than in an isotonic buffer. In addition, a fresh opened vial of water was immediately used to prepare a salt-free diluent (0×). These ionic strength adjusted FSL constructs were applied to MG paper and allowed to be in contact with the surface for between 1 and 1800 s before being washed in PBS pH 7. Binding of the FSL constructs to the surface was then determined by EIA (Figure 10).

Essentially Atri-Ad-DOPE was unaffected by the presence of salts over a large concentration range, with binding to the surface occurring within a second. In the absence of salt Atetra-CMG-DOPE was also able to bind in 1 s, but in the presence of low levels of salt this binding time increased to 15 s. Further increases in salt levels had no effect. These results possibly relate only to MG paper and performance on other surfaces (and with different concentrations of FSL constructs and pH) could potentially produce different results to those obtained on paper.

#### 2.2.4. Effect of Surface and FSL Delivery Solutions

**Figure 11:**
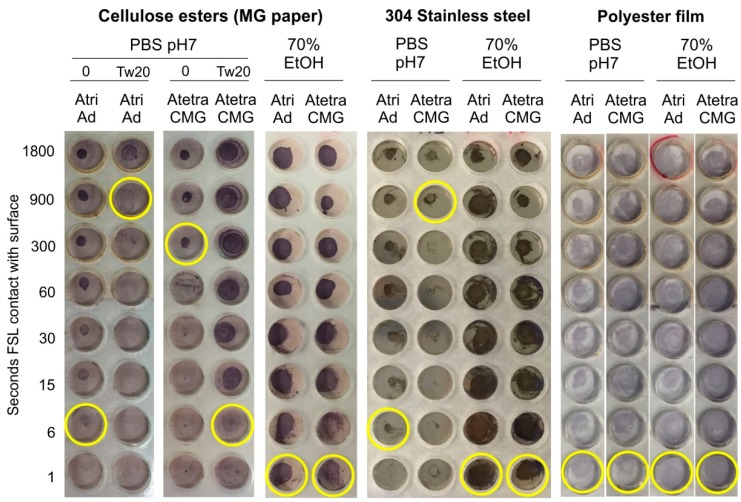
Effect of FSL application buffer (PBS, surfactant (Tw20), 70% ethanol) on the binding characteristics of Atri-Ad-DOPE and Atetra-CMG-DOPE when applied as a 1 µL spots (50 µmol/L) on paper, stainless steel and polyester film. Columns show the time of contact in seconds of the FSL with the surface before being washed away with PBS. The last clearly positive reaction in each column is indicated by a yellow circle.

The influence of the surface type on binding was evaluated by applying a 1 µL spot of 50 µmol/L Atri-Ad-DOPE and Atetra-CMG-DOPE in a pH 7 buffer to three different surfaces (Figure 11). On paper (cellulose ester) and stainless steel the Atri-Ad-DOPE construct produced good positive results at 6 s, however on polyester film a strong positive result was obtained in 1 second. In contrast, Atetra-CMG-DOPE took five min (300 s) to give a clear reaction on paper, and 15 min (900 s) on stainless steel, but only 1 s on polyester film. These results clearly indicate the nature of the surface will have significant impact on the rate at which the FSL is able to bind to the surface.

By adding 0.2% (*v*/*v*) of the surfactant Tween 20 (Tw20) to the PBS application buffer, the ability of Atri-Ad-DOPE to bind to paper was negatively impacted with the binding time changing from 6 seconds to 900 s (15 min). In stark contrast, the addition of Tween 20 to the Atetra-CMG-DOPE positively affected binding and reduced the binding time from 300 to 6 s. The effect of the reduction of surface tension by the surfactant could also be seen with a larger diameter of the spot applied. These results indicate the presence of a surfactant may have significant impact on the rate at which the FSL is able to bind to the surface depending on the type of spacer (and potentially also the surface).

When both constructs were applied in 70% ethanol they were able to form a coating in 1 s on all surfaces. These results suggest that application of the constructs in a solvent is preferable to their interaction and binding with surfaces, presumably because the micelles are less stable.

It should be noted that CMG spacer FSL constructs are not soluble in 96% ethanol (Table 2). Atri-Ad-DOPE is soluble in both 100% methanol and 96% ethanol and both Atetra-CMG-DOPE and Atri-Ad-DOPE constructs were soluble in 100% isopropanol. The observation that tri and tetrasaccharide CMG constructs were equally insoluble in 96% ethanol indicates the spacer was responsible for this effect.

**Table 2:** Solubility of 1 mM Atri-Ad-DOPE, Atri-CMG-DOPE and Atetra-CMG-DOPE in methanol and ethanol.

Solvent	Atri-Ad-DOPE		Atri-CMG-DOPE		Atetra-CMG-DOPE	
	Methanol	Ethanol	Methanol	Ethanol	Methanol	Ethanol
96%	+ ^1^	+	+	P ^2^	+	P
80%	+	+	+	+	+	+
70%	+	+	+	+	+	+

^1^ + = soluble, ^2^ P = precipitate (insoluble).

### 2.3. Stability

#### 2.3.1. Serum

**Figure 12:**
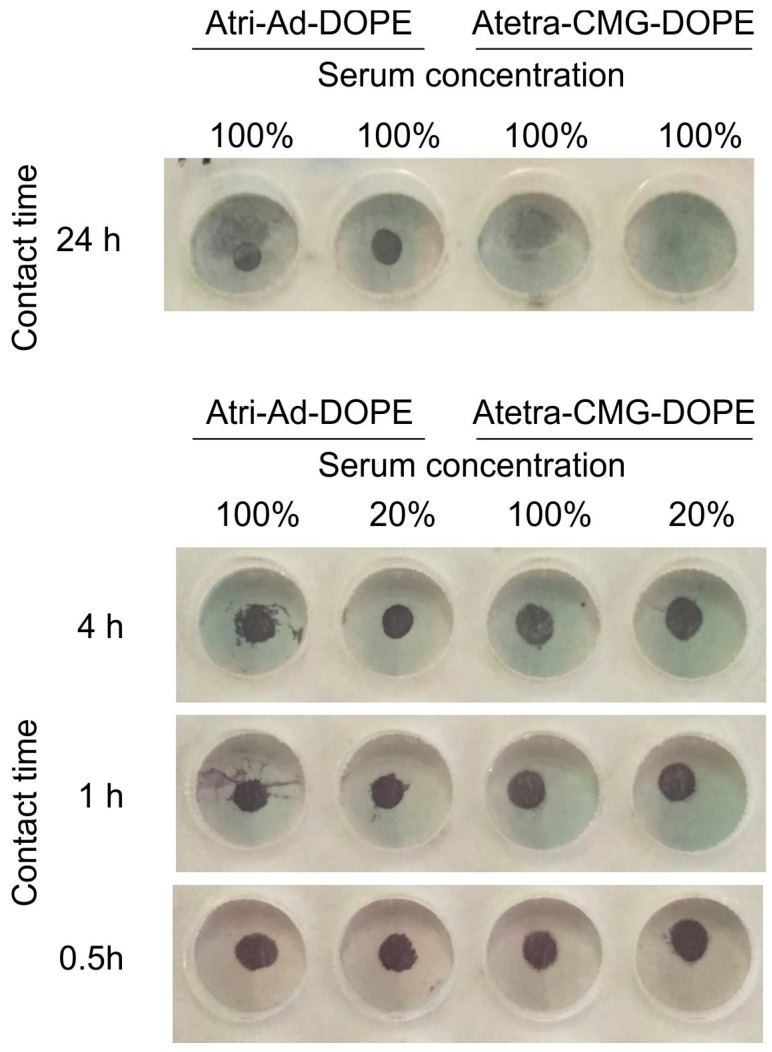
Effect of contact with human serum on the binding characteristics of Atri-Ad-DOPE and Atetra-CMG-DOPE when applied as a 1 µL spots (50 µmol/L) on paper, and exposed to serum for up to 24 h. After exposure to serum the presence of blood group A FSL constructs was determined by EIA with monoclonal anti-A. Serum concentration values relate to 100% being undiluted serum and 20% is serum diluted 1:5.

The need for the glycan coating to be stable in the presence of serum is critical to its applicability to diagnostics assays. To evaluate serum stability we spotted Atri-Ad-DOPE and Atetra-CMG-DOPE onto paper then soaked the spotted membranes in undiluted serum for 24 h. In addition undiluted serum and 1:5 diluted (in PBS) serum was tested against the FSL spots for up to 4 h. Following incubation, plates were washed and tested by EIA with monoclonal anti-A (Figure 12). Group AB serum was selected so as not to contain anti-A, which would interfere with the binding of the monoclonal anti-A (whose purpose was to detect the presence of blood group A FSL constructs). After 24 h of contact with serum the Atri-Ad-DOPE constructs were still present although the Atetra-CMG-DOPE constructs had been mostly removed. Between 0.5 to 4 h contact with serum both Atri-Ad-DOPE and Atetra-CMG-DOPE were clearly present. No differences were noted with diluted serum where lipid loading was reduced to 20% of normal (not tested at 24 h). These results show that both constructs are sufficiently stable on paper membranes to undertake diagnostic assays with up to 4 h incubation against undiluted serum.

#### 2.3.2. Alcohol

**Figure 13:**
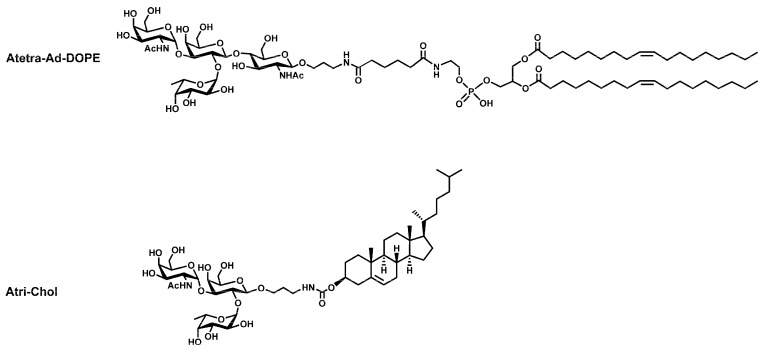
Schematic diagrams of two additional blood group A function-spacer-lipid (FSL) constructs used in this paper. The upper schematic Atetra-Ad-DOPE shows an FSL with a tetrasaccharide blood group type 2 A antigen with an adipate spacer while the lower schematic is of an A trisaccharide with a cholesterol lipid tail (Atri-Chol).

To partially evaluate the strength and mechanisms of binding, printed FSL constructs were exposed to varying concentrations of alcohol. In addition to the two primary constructs Atri-Ad-DOPE and Atetra-CMG-DOPE (Figure 1) two additional constructs Atetra-Ad-DOPE and Atri-Chol (Figure 13) were also available for this analysis.

All four FSL constructs were printed onto paper, dried and then soaked in either water, 50%, 70% or 100% methanol (MeOH) or 50%, 70% or 96% ethanol (EtOH) for 1 h. After exposure, the treated surfaces were dried and assembled into a microplate for EIA immunostaining. Exposure of these printed constructs to water for an hour had no consequence, with all constructs staining strongly by EIA. In contrast, exposure to 50% ethanol or methanol removed almost all variations of blood group A FSL from the paper. Increasing the alcohol concentration to 70% resulted in less efficient removal of Atri-Ad-DOPE (3A0ad) and Atetra-CMG-DOPE (4A2cd), and this was even less effective at >96%. At all concentrations of alcohol the Atri-Chol (3A0as) construct was removed, indicating at least some direct involvement of the lipid tail in the adhesion process. Furthermore the size of the carbohydrate has a role (albeit minimal), as seen in the results observed with 70% EtOH and 100% MeOH, where the more hydrophilic A tetra construct (4A2ad) appeared slightly more easily removed from the surface than was the trisaccharide (3A0ad). The type of the FSL spacer when exposed to 70% EtOH and 100% MeOH had a substantial effect with the tetrasaccharide with the adipate based spacer (4A2ad) being more easily removed than the tetrasaccharide with the CMG spacer (4A2cd). Of particular note was the differences observed with 100% methanol and 96% ethanol, with ethanol much less effective at removing the adipate based FSLs (3A0ad and 4A2ad) than was methanol. This effect was more striking with the tetrasaccharide being more resistant to removal in 96% ethanol than in methanol. These results are supported by the observations that the Atetra-CMG-DOPE and Atri-CMG-DOPE disperse in 100% methanol but are insoluble in 96% ethanol (Table 2).

**Figure 14:**
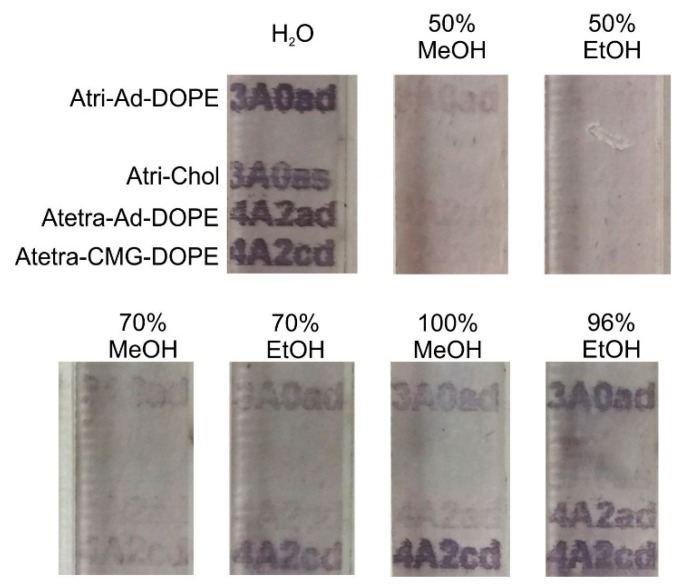
Elution profiles of four blood group A FSL variants printed on paper and exposed to 50%, 70% and 96% ethanol and methanol for 1 h. The FSL variants Atri-Ad-DOPE (3A0ad), Atri-Ad-sterol (3A0as), Atetra-Ad-DOPE (4A2ad) and Atetra-CMG-DOPE (4A2cd) when exposed to water (H_2_O) for 1 h all stained strongly by EIA. Exposure to 50% alcohol was effective at removing most constructs, but increasing concentrations of alcohol were less effective. Differences observed can be partially attributable to the type of lipid tail, spacer and size of the saccharide moiety. These results are probably only valid for this type of surface (paper).

It should be noted that some of these surface effects reported here for paper are surface type dependent and different interactions and stability are observed on different surfaces (e.g., Figure 14).

#### 2.3.3. Detergent/Surfactant

**Figure 15:**
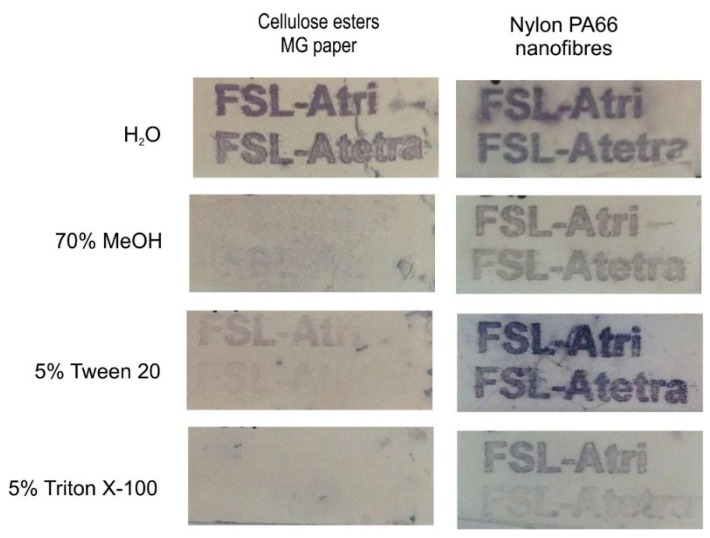
Detergent/surfactant stability of blood group A FSL on paper and nylon nanofibres. FSL variants Atri-Ad-DOPE (printed as FSL-Atri in image) and Atetra-CMG-DOPE (printed as FSL-Atetra in image) were printed on both paper and nylon nanofibres, exposed to deionised water (H_2_O), 70% methanol (MeOH), 5% Tween 20 or 5% Triton X-100 for 1 h, washed then immunostained by EIA.

To further evaluate the strength and mechanisms of binding, printed FSL constructs were exposed to high concentrations of two different routine laboratory detergent/surfactants: Tween 20 (Polyethylene glycol sorbitan monolaurate—a non-ionic detergent) and Triton X-100 (Polyethylene glycol *tert*-octylphenyl ether—a non-ionic surfactant). Two different FSL variants were available for evaluation: Atri-Ad-DOPE and Atetra-CMG-DOPE. Both FSL constructs were printed onto paper or nylon PA66 nanofibres then soaked in either water, 70% methanol or 5% Tween 20 or 5% Triton X-100 for 1 h, then washed and stained by EIA (Figure 15). The 70% methanol results on paper correlated with previous results of significant elution from the paper surface, however 70% methanol removal of FSLs was much less effective on the nylon nanofibres. The 5% Tween 20 solution was able to remove most of the Atetra-CMG-DOPE constructs from paper but was less effective at removing the Atri-Ad-DOPE constructs. Concentrations of Tween 20 less than 1% did not have any effect on removal of Atri-Ad-DOPE from paper (results not shown). In contrast, soaking the FSL printed nylon membrane in 5% Tween 20 appeared to either have no effect, or possibly slight enhancement of the immunoreactivity of the FSL constructs present.

The surfactant Triton X-100 in contrast was able to effectively remove both constructs from paper (even with concentrations as low as 0.05%—not shown) however it was much less effective in removal of FSL constructs from nylon nanofibres. The adipate spacer FSL, Atri-Ad-DOPE (Figure 15—FSL-Atri) was also more resistant to removal than the CMG spacer construct on nylon nanofibres. Blood group A FSL was removed from polyester film with all concentrations of alcohol and both detergents.

Together these results clearly indicate variability of the stability of different FSL coatings on different surfaces and the need to experimentally determine and optimise each construct for each surface.

## 3. Experimental Section

### 3.1. Synthesis of FSL Constructs

The FSL glycans described in the paper, and other related FSL glycans, and their synthesis are described in detail elsewhere [4,5,8].

### 3.2. Surfaces

#### 3.2.1. Disc Coupons

Disc coupons for CDC and RDR reactors (Figure 2) were used as defined surface materials and were obtained from BioSurface Technologies Corporation (Bozeman, MT, USA). Coupons were labelled by placing a 1 µL drop of FSL (50 µmol/L in water) at either the 12 o’clock position (Atri-Ad-DOPE) or 6 o’clock position (Atetra-CMG-DOPE) and allowed to air dry. Coupons were washed 3× with PBS before visualisation of the FSL by EIA.

#### 3.2.2. Membranes and Planar Surfaces

A variety of filtration membranes and planar surfaces including metals and papers (Figure 4) were obtained from different sources. Filtration membranes were obtained from Sterlitech, USA and Millipore, France; TLC plates (Alugram SIL G/UV silica gel 60, and C18 RP-18W) from Macherey-Nagel, Düren, Germany; microspheres from Nanomi B.V., Oldenzaal, The Netherlands and Millipore, Pithiver, France; gold and silver foils from The Gold Leaf Factory International, Seaford, Vic, Australia; silver dressing from Silverlon, Argentum Medical, Geneva, IL, USA; and 24 varieties of coated and uncoated papers from Spicers Paper, Auckland, New Zealand. Membranes and planar surfaces were labelled with inkjet printer delivered FSL constructs and/or by direct application (dipping, spotting or paintbrush). Nanofibres for printing were supplied by Revolution Fibres, Auckland, New Zealand.

#### 3.2.3. Electrospinning Nanofibres Incorporating FSL Constructs

Nanofibres incorporating FSL constructs were prepared using an in house apparatus at the premises of Revolution Fibres Ltd., Auckland, New Zealand [11]. Dispersions of polyvinyl butyral (PVB) were prepared at a concentration of 10% (*w*/*v*) in 100% ethanol. Dispersions of cellulose acetate (CA) were prepared at a concentration of 17% (*w*/*v*) in 70:15:15 (*v*/*v*/*v*) MEK/DMA/DMF. Mats of nanofibres were electrospun from these dispersions with or without the addition of Atri-Ad-DOPE. Where blood group A FSL constructs were added to the dispersions 50 μL of a 10 mg/mL solution of the construct in 100% ethanol was added to a 5 mL volume of the dispersion of the polymer to provide a final concentration of 100 μg/mL. The speed of rotation, the distance to the collector plate and voltage were adjusted to optimise deposition of the nanofibres electrospun from each of the dispersions. Spun fibres twisted into threads were immobilized at one end with 80 micron document laminating film then immunostained by the EIA technique.

### 3.3. Methods for FSL Coating Surfaces

#### 3.3.1. Direct Application

The easiest and fastest approach to applying an FSL to a surface is by simple contact. A variety of direct application methods were available including applying the construct as a 1 µL spot to a surface with a multi-pipette, or application with a paintbrush, or dipping into a solution or flooding the surface. Each variation including surface type, FSL concentration, buffer, drying rate, *etc*., has the potential to create a slightly different coating by affecting the way the constructs assembles at the surface [4]. Unless stated otherwise the usual concentration for direct application of FSL constructs was at a concentration 50 µmol/L.

#### 3.3.2. Inkjet Printing

FSL constructs for printing were prepared as 600 µmol/L solutions in PBS containing 0.25% bromophenol blue (a visualisation dye which is lost during incubation) [8]. An alphanumeric code representing each FSL construct or the name of surface the FSL was being modified (Figure 3, Figure 4, Figure 5, Figure 12, Figure 14 and Figure 15) was printed with an Epsom stylus T21 inkjet printer where the inkjet cartridge was loaded with the appropriate FSL construct. Printer heads and cartridges were thoroughly cleaned with 70% methanol followed by PBS and blank printing between experiments.

### 3.4. Microplate Assembly

Where appropriate to allow multiple reactions microplates were prepared either with surfaces pre-printed with FSL constructs (Figure 3) or unmodified for later spot application of FSL constructs (Figure 8, Figure 9, Figure 10 and Figure 11). Fibrous surfaces such as paper were first prepared by using a laser cutter (X660 Universal laser Systems, Scottsdale, AZ, USA) to cut reaction well shapes from one side of an 80 micron document laminating film (to exclude laminating over the printed FSLs) which was then laminated (Document Laminator, GBC 1200, Lake Zurich, IL, USA) onto the either FSL printed surface or a blank surface, leaving the reaction areas exposed. This step, which both stabilizes the membrane and prevents leakage between microwells was not required for non-fibrous surfaces such as stainless steel or polyester film. A 2 mm acrylic (Clear Cast Acrylic, Modern Plastics, Auckland, New Zealand) template covered with one-piece of double-side adhesive sheet was then laser cut to shape and adhered to the surface, to create reaction wells (Figure 3). Prepared plates were stored at room temperature until ready for use.

### 3.5. Visualisation of Blood Group A FSL Constructs

#### 3.5.1. Enzyme Immunoassay (EIA)

FSL coated surfaces were first blocked with BSA-PBS (2% bovine serum albumin in phosphate buffered saline, pH 7.2) for 30 min then decanted. Each surface or prepared microplate well was flooded or filled with IgM monoclonal anti-A (Epiclone anti-A, Cat# 0261, CSL, Seaford, VIC, Australia) diluted 1:4 with BSA-PBS and then incubated for 30 min at RT. This anti-A reagent has been previously established to show specific binding against the blood group A antigen, including in the form of FSL constructs [4,5,7,8,10,11]. Unprinted areas within the assay are internal blanks.

Microplates and surfaces were washed 6 times by immersion for 20 s in beakers of fresh PBS. Each microwell or surface was filled or flooded with goat anti-mouse total immunoglobulin conjugated to alkaline phosphatase (Millipore, Temecula, CA, USA) diluted 1:1000 in 2% BSA-PBS and incubated for 30 min at room temperature before being washed 6 times in PBS. Precipitating chromogenic substrate (NBT/BCIP stock solution, 18.8 mg/mL nitro blue tetrazolium chloride and 9.4 mg/mL 5-bromo-4-chloro-3-indolyl phosphate toluidine salt in 67% DMSO (*v*/*v*); Roche, Mannheim, Germany) was diluted 1:50 in substrate buffer (100 mM Tris, 100 mM NaCl, 50 mM MgCl_2_, pH 9.5) and added to each microwell or flooded onto surfaces. Development of reactions at RT occurred within 4 minutes and the reaction was stopped by rinsing with water. Developed reactions were allowed to air dry and could be stored unprotected at RT. Caution: the ability of the NBT/BCIP precipitate to be retained on the surface can significantly influence the interpretation of the result.

#### 3.5.2. Cell Binding via Antibody Bound to FSL

An alternative method to visualisation by EIA was to allow the binding of cells to the surfaces via IgM antibody. FSL modified membranes were blocked by flooding with BSA-PBS (2% bovine serum albumin in phosphate buffered saline, pH 7.2) for 60 min then decanted. Surfaces were then flooded with IgM monoclonal anti-A (Epiclone anti-A, Cat# 0261, Seaford, CSL, Australia) diluted 1:10 with BSA-PBS for 1 h. Surfaces were then washed 6 times by immersion for 20 s in beakers of fresh PBS. Each washed membrane was then flooded with a 2% suspension of washed blood group A red cells and allowed to incubate for 1 h (incubation allows the red cells to settle onto the surface). Membranes were very gently washed by flooding the surface with PBS (from a pipette away from the surface) until the unbound cells were cleared. The adhesion of the cells is very fragile on most surfaces (except cotton) and gentle washing is essential.

An alternative method is to apply the constructs onto microspheres, and then attach antibody to the microspheres. Provided antibody is added in excess the beads will be unable to cross-link. When washed these blood group A FSL anti-A coated microspheres can then be used to capture blood group A cells.

### 3.6. Scanning Electron Microscopy (SEM)

An Hitachi SU-70 Analytical Field Emission scanning electron microscope (SEM) (Tokyo, Japan) was used to capture surface images after being sputter coated with platinum for 60 s (15 keV accelerating voltage). SEM analysis of surfaces with cells were first treated by flooding with 0.5% glutaraldehyde in PBS for 20 min and then gently washed with water.

## 4. Conclusions

Experimental data is clearly able to show the potential of FSL constructs when optimised to rapidly (within 1 s) coat almost any non-biological surface. However this labelling and its stability are clearly variable dependent. In order to achieve optimal and rapid labelling, the type of constructs used (spacer, lipid and functional head size), the method of application onto a surface (concentration, diluent, pH, ionic strength, surfactant, solvent, *etc*.) should all be determined and optimised for each surface intended to be modified. It is of note that most variability was only observed with contact times less than 5 min, and if a 30 min FSL contact time was used, then most of these variable dependent interactions would not be observed. Currently dynamic light scattering, atomic force microscopy, and other physical methods are in progress to allow us to determine detailed FSL surface layer architectures; which will assist in determining optimal coating conditions.

Physical adsorption of FSL constructs to surfaces although not a permanent modification are sufficiently robust to be suitable for enzyme immunoassays, ligand-receptor and cellular/biological interactions. Together with the ability to be able to use the same or different constructs to modify cells and viruses [4,5] and simultaneously use multiple FSL constructs, including non-glycan FSL variations [7], FSL modification of surfaces is a useful alternative and additive technology to existing covalent glycosylation methods.
